# Temperature-Dependent Effects of Hydroxyethyl Methyl Cellulose on Rheological Properties and Microstructural Evolution of Robotic Plastering Mortars

**DOI:** 10.3390/ma18204664

**Published:** 2025-10-10

**Authors:** Guangjie Ling, Hongbin Yang, Sifeng Liu

**Affiliations:** 1 School of Materials Science and Engineering, Tongji University, Shanghai 201804, China; ling_guangjie@tongji.edu.cn; 2 Shanghai Weibuild Technology Co., Ltd., Shanghai 200949, China; hongbin.yang@we-i-build.com; 3 Key Laboratory of Performance Evolution and Control for Engineering Structures (Ministry of Education), Tongji University, 1239, Siping Rd., Shanghai 200092, China

**Keywords:** hydroxyethyl methyl cellulose, rheology, robotic plastering mortar, temperature, ultrasonic pulse velocity

## Abstract

Temperature-induced instability in early-age rheology poses a major challenge to the pumpability and application of robotic plastering mortars. This study systematically investigates the temperature-dependent effects of a high-viscosity (75,000 mPa·s) hydroxyethyl methyl cellulose (HEMC) on the rheological properties and early microstructural evolution of mortars at 5 °C, 20 °C, and 40 °C. Mortars with HEMC dosages from 0 to 0.25 wt% were tested using rheological measurements, ultrasonic pulse velocity (UPV), and complementary microstructural analyses (XRD, FTIR, and SEM–EDS). Results show that HEMC reduced the initial static yield stress while monotonically increasing plastic viscosity, with the thickening effect more pronounced at higher temperatures. Notably, at 40 °C, the initial plastic viscosity of a 0.25% HEMC mix reached 14.4 Pa·s, a 133% increase compared to the control group. HEMC also effectively retarded the time-dependent increase in yield stress and stabilized plastic viscosity, thereby mitigating the adverse influence of elevated temperature. UPV confirmed that HEMC delayed microstructural formation, in agreement with the observed retardation of hydration reactions. At 40 °C, a 0.10% HEMC dosage postponed the percolation threshold from 67 min to 150 min, highlighting its strong retardation effect. Microstructural tests further revealed that HEMC delayed CH formation, refined C–S–H gels, and reduced the crystallinity of AFt, supporting the rheological and ultrasonic findings. A statistically significant, moderate-to-strong correlation (r = 0.88, R^2^ = 0.77, *p* < 0.001) was established between static yield stress and UPV, indicating that macroscopic rheological resistance responds to microstructural evolution. Based on these results, the recommended HEMC dosages to achieve stable rheological performance are 0.05–0.10% at 5 °C, 0.10–0.15% at 20 °C, and 0.15–0.20% at 40 °C.

## 1. Introduction

Robotic plastering represents an innovative, automated method that employs systematic, digital control [1,2,3]. This process involves the robot’s actuator activating a mortar pump to apply the material with a consistent compressive force of 20–30 kgf against a wall. This unique application method necessitates tightly controlled rheological properties for robotic plastering mortars, ensuring reliable pumpability and substrate adhesion. The key distinction between these and traditional cement mortars lies in the critical timeframe for rheological stability. Based on practical feedback from construction sites, robotic plastering mortars must maintain stable rheological properties for at least two hours to ensure continuous operation, a requirement that is not explicitly specified for traditional plastering mortars. Given this runtime, these systems must sustain consistent flow characteristics under mechanical pressure while rapidly developing sufficient stiffness after application to prevent sagging or slippage. Among the various factors influencing these properties, environmental temperature fluctuations are a major challenge, as they can significantly disrupt the stability of flow behavior and setting performance [4,5,6,7].

For plastering mortars, the rheological behavior is principally governed by the evolving microstructure due to cement hydration and significantly influenced by temperature [8,9,10]. During the early stages, the hydration products are gradually formed and interconnect to establish a structural network, leading to an increase in yield stress and plastic viscosity [11,12]. In addition to this gradual development, temperature acts to accelerate or retard hydration kinetics: at low temperatures (e.g., 5–10 °C), hydration rates are reduced, extending the dormant period and delaying the development of flow resistance [4,13], whereas at elevated temperatures (e.g., 30–40 °C), accelerated hydration results in shortened open time and rapid stiffening [4,14,15,16]. Therefore, this study selected 5 °C, 20 °C, and 40 °C as representative temperatures to simulate typical cold, standard room temperature, and hot construction environments, respectively. The combined influence of time and temperature highlights the necessity of understanding how temperature modulates the temporal evolution of rheological properties in order to ensure consistent mortar performance under field conditions.

In order to mitigate time- and temperature-induced variations in rheological behavior, HEMC has been employed as a viscosity-modifying admixture [10,16,17,18,19]. When added to mortar, HEMC adsorbs onto cement particles, forming a three-dimensional polymer network that binds water through hydrogen bonding [20,21,22,23]. This action consequently alters the mortar’s rheological characteristics and retards cement hydration [8,24,25]. The magnitude of these effects has been found to depend directly on the molecular weight of the polymer, with higher molecular weight HEMC producing more pronounced thickening [19,26]. To address the critical anti-sagging requirements of robotic plastering, this study focuses on a representative high-viscosity grade (75,000 mPa·s) to investigate its temperature-dependent behavior. Chen et al. [27] observed that, at 20 °C, high-viscosity HEMC with extended polymer chains produced increased static yield stress and reduced plastic viscosity in ultra-high-performance concrete, while also exhibiting superior rheological stability under elevated temperature. Furthermore, Pichniarczyk and Niziurska [28] demonstrated that, at ambient temperature and a viscosity grade of 65,000–75,000 mPa·s, methyl cellulose reduced mortar slip by 85% without compromising open time.

Temperature not only affects cement hydration but also modulates HEMC performance. Gu et al. [29] found that, above 40 °C, HEMC’s water retention efficiency declines as its apparent viscosity and hydrodynamic radius decrease, while cement hydration is accelerated. Their work further delineates two temperature-dependent regimes: below 40 °C, rheological behavior is dominated by HEMC characteristics—such as viscosity, adsorption, and conformation—whereas, above this threshold, accelerated hydration reactions become the predominant factor. Furthermore, Lim et al. [30] demonstrated that elevated temperatures lead to the swelling of cellulose ether long-chain molecules. This swelling exposes more hydrogen bonding sites while simultaneously reducing the stability of hydrogen bonds between cellulose ethers and water molecules, consequently reducing solution viscosity.

To link rheological changes with microstructural evolution, ultrasonic pulse velocity (UPV) testing is adopted as a real-time, non-destructive technique [11,31,32,33,34,35]. Ye et al. [36] observed that during the initial dormant stage of cement hydration, UPV values remain low (200–400 m/s), since ultrasonic waves traverse the primarily liquid suspension. As hydration progresses and solid phases begin to interconnect—reaching the percolation threshold—a clear inflection appears in the UPV curve. This inflection marks the start of rapid velocity increases, reflecting the formation of a continuous solid network and concurrent rises in yield stress and viscosity [11].

Although the influence of temperature and cellulose ethers has been well documented, limited data are available on the coupled effects of high-viscosity HEMC on the rheology and microstructure of robotic plastering mortars. The present study addresses this gap by evaluating the combined effects of temperature, time, and HEMC with a viscosity of 75,000 mPa·s on rheological properties and microstructural evolution. Rheological tests were complemented with ultrasonic pulse velocity (UPV) monitoring and microstructural analyses (XRD, FTIR, and SEM–EDS) of mortars at 5 °C, 20 °C, and 40 °C during the first two hours of hydration. Such data are essential to clarify the mechanisms of workability retention in robotic plastering mortars under different environmental conditions.

## 2. Materials and Methods

### 2.1. Materials

**Figure 1:**
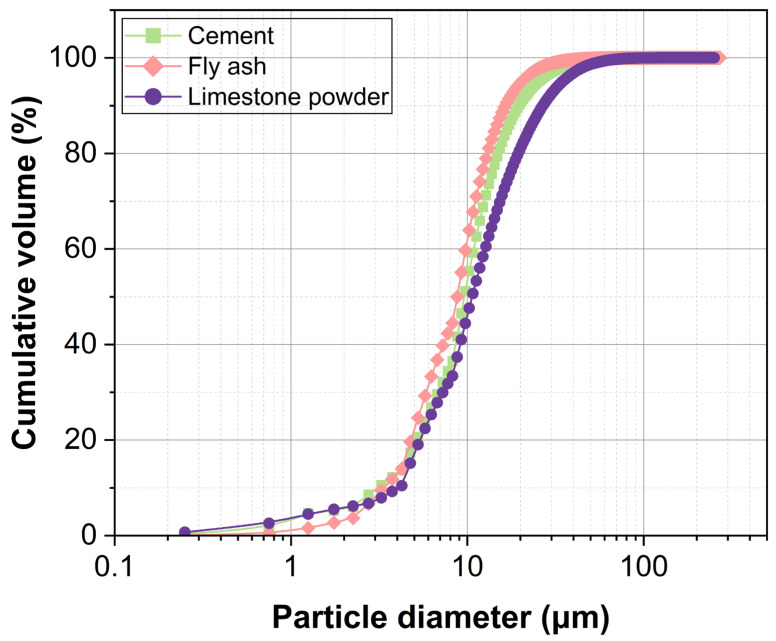
Particle size distribution of cement, fly ash, and limestone powder.

The HEMC used in this study was a commercial cellulose ether with a viscosity of 75,000 mPa·s, supplied by Tianpu Chemicals Co., Ltd., Zhangjiagang, China. Ordinary Portland cement (P·O 42.5) from Hefei, China, conforming to Chinese standard GB 175-2023 [37] was employed; its chemical composition is listed in Table 1. Fly ash (Jiaxing, China) and limestone powder (Langfang, China) were incorporated as mineral admixtures to enhance mortar flowability while reducing production costs. The chemical composition of fly ash is presented in Table 1, while the limestone powder, with a purity of over 95% calcium carbonate (CaCO_3_), was also used. The particle size distributions of cement, fly ash, and limestone powder are shown in Figure 1. Manufactured sand (Langfang, China) was used as the aggregate; its gradation and physical properties are given in Table 2 and Table 3, respectively. Tap water was used for mixing.

### 2.2. Methods

#### 2.2.1. Sample Preparation

As shown in Table 4, six mortar mixtures with varying HEMC dosages were prepared. The HEMC dosages were set at 0, 0.05, 0.10, 0.15, 0.20 and 0.25% by mass of binder. The mixtures had a sand-to-binder ratio of 3:1, with the binder consisting of cement, 20% of which was replaced by fly ash. Based on preliminary experiments, fly ash was used as a reactive supplementary cementitious material to enhance flowability, while 10% of the sand was replaced by limestone powder to improve particle packing. To ensure a consistent initial workability and to isolate the effects of HEMC, the water content of each mixture was adjusted to achieve an initial flow diameter of 185.0 ± 5.0 mm, measured via a flow table test. This specific flow range was selected based on construction site feedback, as it is critical for smooth pumpability and plastering.

**Figure 2:**
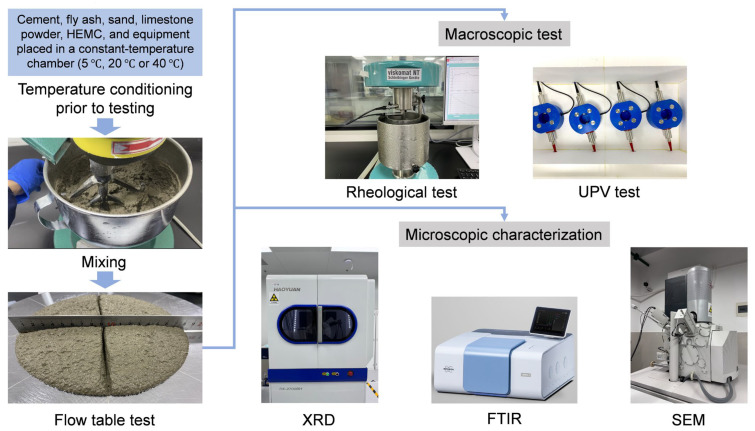
Flowchart of mortar sample preparation and testing procedures.

Figure 2 presents the detailed flowchart of the sample preparation and testing procedures, along with images of the key apparatus. A standardized procedure was followed to ensure consistent shear history and strict temperature control. All mixtures were produced in the same 5 L planetary mixer (Wuxi, China), conforming to Chinese standard GB/T 29756-2013 [38], following an identical mixing sequence (180 s dry mixing, 60 s low-speed mixing with water, 60 s pause, and a final 60 s low-speed mixing). Given the importance of temperature, all materials and equipment were pre-conditioned for two hours in an environmental chamber at the target temperature before mixing. After the flow table test, samples were immediately sealed and returned to the chamber. The rheological tests were performed using a rheometer with an internal water-circulated temperature control unit for precise temperature control, while UPV measurements were conducted with samples kept in a custom-built insulated box with real-time temperature monitoring.

For SEM–EDS analysis, mortar fragments were taken at 120 min of hydration, avoiding large sand particles. For XRD and FTIR, corresponding cement pastes with the same binder-to-water ratio and admixture proportions (but without sand) were prepared. At 120 min of hydration, all microstructural samples were immersed in pre-conditioned isopropanol from Shanghai, China, which was cooled or heated to the target temperature to stop hydration, with solvent exchange performed three times for 10 min each. The samples were then vacuum-dried for 24 h. For XRD and FTIR, the dried pastes were subsequently ground manually in an agate mortar to obtain fine powders.

#### 2.2.2. Rheological Tests

Rheological measurements were carried out on a Schleibinger Viskomat NT mortar rheometer (Buchbach, Germany) fitted with a water-circulated temperature control unit. The key parameters of the rheometer were a sample volume of 370 mL and a fishbone spindle with a diameter of 68 mm. To ensure the reliability and repeatability of the measurements, a preliminary experiment was conducted on the 20 °C, 0.05% HEMC, 10 min sample. Three replicates were tested to assess the reproducibility of the rheological measurements, and the results for static yield stress, dynamic yield stress, and plastic viscosity are presented in Table 5. The low coefficients of variation (CV) for all parameters—1.3% for static yield stress, 5.4% for dynamic yield stress, and 1.8% for plastic viscosity—demonstrate the high reproducibility of the experimental setup and the stability of the mortar samples. These results confirm the overall reliability of the rheological data presented in this study. Mortar samples were tested at 10, 60, and 120 min after water addition. After each measurement, the already sheared sample was discarded to ensure a consistent initial shear history for all subsequent test samples.

(1) Static shear test

**Figure 3:**
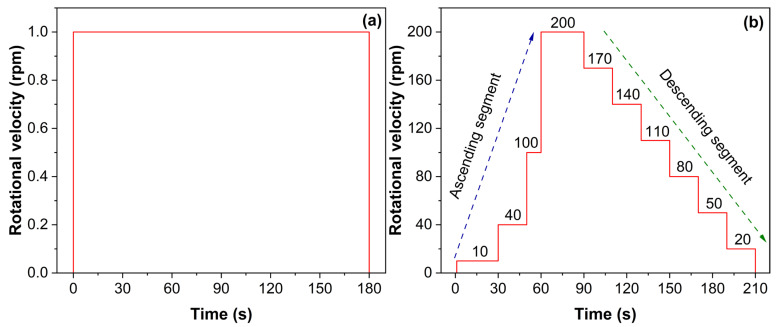
Shear test protocols: (**a**) static shear; (**b**) dynamic shear.

Static yield stress ($\tau_{i}$) and equilibrium stress ($\tau_{e}$) were measured using a constant shear-rate protocol (1 rpm, corresponding to 0.335 s^−1^) under controlled temperature. Mortar was sheared continuously for 180 s at 1 rpm (Figure 3a). The shear stress–time curve exhibits (1) a rapid rise to a peak stress $\tau_{i}$—defined as the static yield stress at which the mortar’s microstructural network fails—and (2) a subsequent decay to a steady stress $\tau_{e}$, representing the residual structural resistance under flow. The difference between $\tau_{i}$ and $\tau_{e}$ reflects the extent of microstructural breakdown and thixotropic behavior.

(2) Dynamic shear test

Flow curves were obtained using a combined ascending-descending shear-rate protocol (Figure 3b). First, an ascending sequence (10, 40, 100, 200 rpm for 30, 20, 10, and 30 s, respectively) was applied to ensure complete breakdown of the mortar microstructure. Next, the descending sequence reduced the shear rate in 30 rpm steps down to zero, holding each step for 20 s to achieve equilibrium stress at each rate. For the rheological curve fitting, data points from the descending segment, specifically from t = 71 s to 210 s, were selected. Three established rheological models—the Bingham, Modified Bingham, and Herschel–Bulkley models—were initially used to fit the data. The optimal model was selected by evaluating the coefficient of determination (R^2^) and the Akaike Information Criterion (AIC) for each fit. Although all three models showed good correlation, the Bingham model was ultimately chosen as it consistently provided a high goodness-of-fit (R^2^ > 0.95 for most cases) while being the most parsimonious model with physically meaningful parameters. Therefore, all rheological data were analyzed using the Bingham model:

(1) $$\tau =\tau_{0}+\eta_{p}\dot{\gamma }$$

where τ (Pa) is shear stress, $\tau_{0}$ (Pa) is dynamic yield stress, $\eta_{p}$ (Pa·s) is plastic viscosity, $\dot{\gamma }$ (s−1) is shear rate.

#### 2.2.3. UPV Test

Ultrasonic pulse velocity and acceleration were monitored continuously with an IP-8 Ultrasonic Transmission System (UltraTest GmbH, Achim, Germany). Immediately after mixing, fresh mortar was cast into a silicone mold (Φ 50 mm × 50 mm), and the ultrasonic transmitter and receiver were mounted directly opposite at a fixed separation of 40 mm. A thermocouple probe inserted into the mold wall recorded the sample temperature once per minute.

Before each test, the system was calibrated using a fused-quartz reference block to ensure measurement accuracy. Tests were conducted at target temperatures of 5 °C, 20 °C, and 40 °C, with the entire assembly—including mold, transducers, and electronics—housed in a temperature-controlled chamber. Ultrasonic pulses were emitted once per min; transit times were converted to UPV by dividing the known path length by the measured time, while acceleration data were calculated by the instrument’s proprietary software during each measurement cycle. Continuous monitoring over 180 min yielded time-resolved profiles of both UPV and acceleration, which enabled the analysis of the early-age microstructural evolution.

#### 2.2.4. XRD Characterization

Powder X-ray diffraction (XRD) was performed on paste samples at 120 min of hydration using a DX-2700BH diffractometer (Haoyuan Instrument, Dandong, China) with Cu Kα radiation (λ = 1.5406 Å, 40 kV, 40 mA). Data were collected over 5–70° (2θ) with a step size of 0.02° and scan speed of 2°/min. Phase identification was carried out using MDI Jade 9 with the ICDD PDF database.

#### 2.2.5. FTIR Characterization

Fourier transform infrared (FTIR) spectra of dried paste samples at 120 min were recorded on an INVENIO spectrometer (Bruker, Ettlingen, Germany) with an ATR accessory. Measurements were collected over 400–4000 cm^−1^ at 4 cm^−1^ resolution with 32 scans, and spectra were baseline-corrected before analysis.

#### 2.2.6. SEM–EDS Characterization

The microstructure and composition of mortar fragments at 120 min were examined by field-emission SEM (Quanta 200F, FEI, Hillsboro, OR, USA) equipped with EDS. Samples were solvent-exchanged, dried, gold-coated, and imaged at 10 kV under high vacuum. EDS point analyses were performed at 18 kV to determine local elemental composition.

## 3. Results and Discussion

### 3.1. Static Rheological Properties

**Figure 4:**
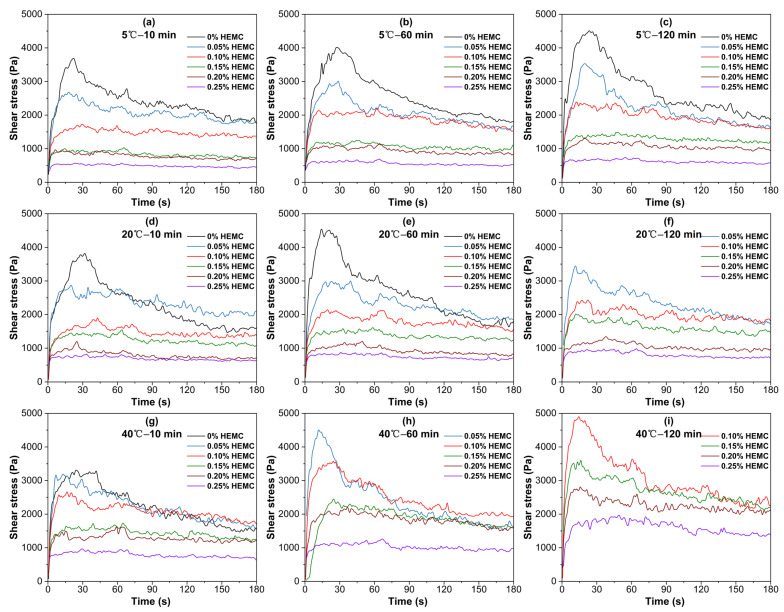
Static shear stress–time curves for mortars with different HEMC dosages, measured at various temperatures and elapsed times: (**a**) 5 °C—10 min; (**b**) 5 °C—60 min; (**c**) 5 °C—120 min; (**d**) 20 °C—10 min; (**e**) 20 °C—60 min; (**f**) 20 °C—120 min; (**g**) 40 °C—10 min; (**h**) 40 °C—60 min; (**i**) 40 °C—120 min.

Figure 4 illustrates the static shear stress–time curves for mortars containing 0, 0.05, 0.10, 0.15, 0.20, and 0.25% HEMC at 5 °C, 20 °C, and 40 °C, recorded at 10, 60, and 120 min under a constant shear rate (1 rpm). Each curve exhibits a peak stress, defined as the static yield stress at structural failure, followed by a gradual decline to an equilibrium stress, indicative of residual structural resistance.

Both the peak and equilibrium stresses decrease as HEMC dosage increases. When HEMC dosage reaches a threshold (≥0.10% at 5 °C and 20 °C; ≥0.15% at 40 °C), the characteristic stress peak disappeared, demonstrating reduced thixotropic response and enhanced structural stability. With prolonged elapsed time, the reappearance of a subtle peak in low-HEMC specimens reflects the ongoing structural development driven by cement hydration, particularly under higher temperatures. Furthermore, the progression of shear stress over time is strongly temperature-dependent. At 5 °C, even the unmodified mortar exhibited slow stress growth. At 20 °C, the unmodified mortar hardened beyond measurement limits at 120 min, whereas HEMC-modified mortars maintained measurable rheology. At 40 °C, all samples showed rapid stress increase, with lower-dosage mortars hardening earlier. Higher HEMC dosages (≥0.15%) consistently moderate the rate of stress build-up over time, confirming the retardation effect on hydration-induced network formation.

**Figure 5:**
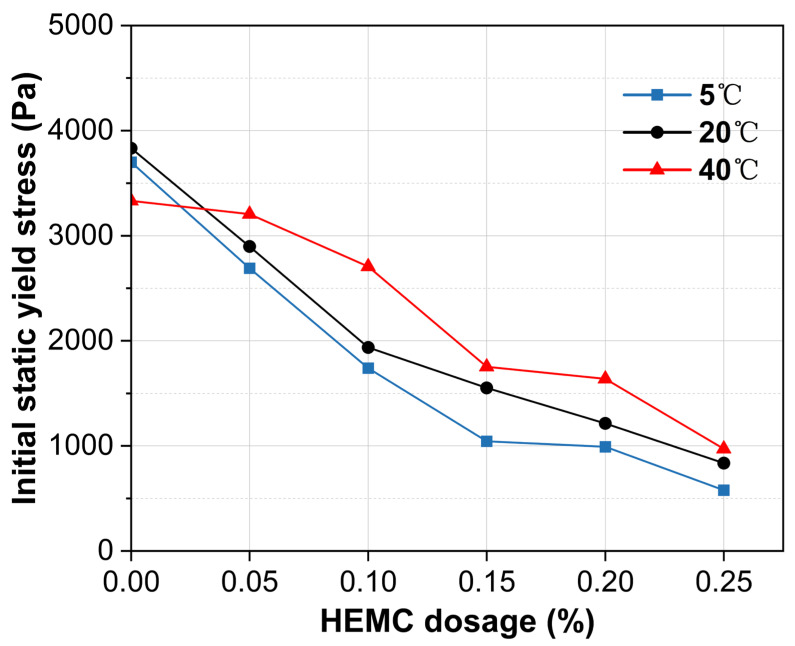
Initial static yield stress (at 10 min) of mortars with different HEMC dosages at 5 °C, 20 °C, and 40 °C.

The initial static yield stress, measured at 10 min, is predominantly influenced by HEMC dosage (Figure 5). A clear trend shows that the initial static yield stress decreases substantially as the HEMC content increases across all tested temperatures. Compared to the mortar without HEMC, the addition of 0.25% HEMC reduced the initial static yield stress by over 70%. This reduction is attributed to the thickening effect of HEMC on the liquid phase, which enhances particle lubrication and encapsulation, thereby lowering the inter-particle static friction that must be overcome to initiate flow [22].

Temperature also modulates the initial static yield stress, though its effect is less pronounced than that of HEMC. For the mortar without HEMC, the initial static yield stress at 40 °C was substantially lower than at 5 °C and 20 °C, likely due to increased bleeding at elevated temperatures which raises the effective water content [29]. Conversely, in HEMC-modified mortars, the polymer’s high water retention counteracts bleeding. As a result, the initial static yield stress in these mortars tends to increase with temperature, and the differences across the temperature range narrow significantly. Notably, at a dosage of 0.25% HEMC, the yield stress values at all three temperatures converge, with variations of less than 30%. This demonstrates that a sufficient dosage of high-viscosity HEMC can effectively buffer the system against temperature-induced variations in initial yield stress.

**Figure 6:**
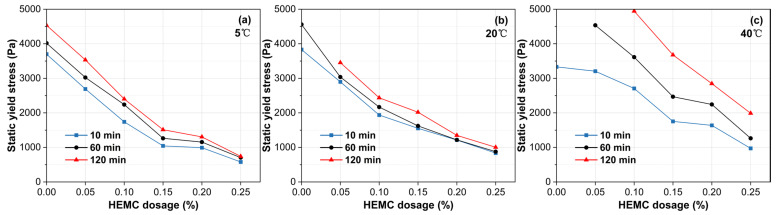
Evolution of static yield stress over time for mortars with different HEMC dosages at: (**a**) 5 °C, (**b**) 20 °C, (**c**) 40 °C.

As illustrated in Figure 6, the static yield stress of all mortars grows over time, reflecting the continuous evolution of the internal microstructure. This structural build-up is significantly accelerated by temperature and retarded by HEMC addition. For the mortar without HEMC, the build-up was slow at 5 °C, but accelerated dramatically at higher temperatures, with the static yield stress exceeding the instrument’s limit of 5000 Pa at 120 min and 60 min for 20 °C and 40 °C, respectively. In contrast, HEMC effectively moderated this process. For HEMC-modified mortars at temperatures of 20 °C and below, the static yield stress increased slowly, with build-up rates remaining below 7.7 Pa/min over 120 min. At 40 °C, the build-up was faster; the rate was 26.6 Pa/min for the 0.05% HEMC mortar. However, increasing the HEMC dosage progressively suppressed this acceleration, with the build-up rate decreasing to just 9.3 Pa/min at a dosage of 0.25% HEMC. These opposing effects confirm that under high-temperature conditions, a higher dosage of HEMC is essential to control the rate of structural build-up and maintain sufficient open time.

### 3.2. Dynamic Rheological Properties

**Figure 7:**
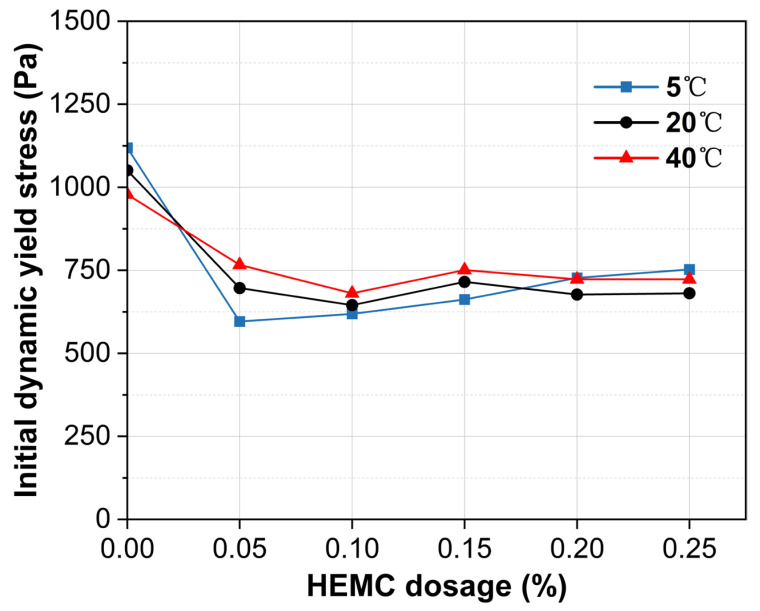
Initial dynamic yield stress (at 10 min) of mortars with different HEMC dosages at 5 °C, 20 °C, and 40 °C.

Figure 7 shows the initial (10 min) dynamic yield stress of mortars containing 0–0.25% HEMC at 5 °C, 20 °C, and 40 °C. The impact of both HEMC and temperature on dynamic yield stress is less pronounced than on static yield stress. This is because dynamic yield stress reflects the critical stress for transitioning from flow to rest and depends on the residual microstructure after shearing. As HEMC dosage increases, dynamic yield stress decreases until a plateau is reached. At 5 °C, the stress reaches a plateau at dosages of 0.05% HEMC and higher. At both 20 and 40 °C, it stabilizes for HEMC dosages of 0.10% or more, yielding plateau values between 625 and 750 Pa. This behavior suggests that beyond these dosages, the formed polymer network fully dominates the residual structural strength.

**Figure 8:**
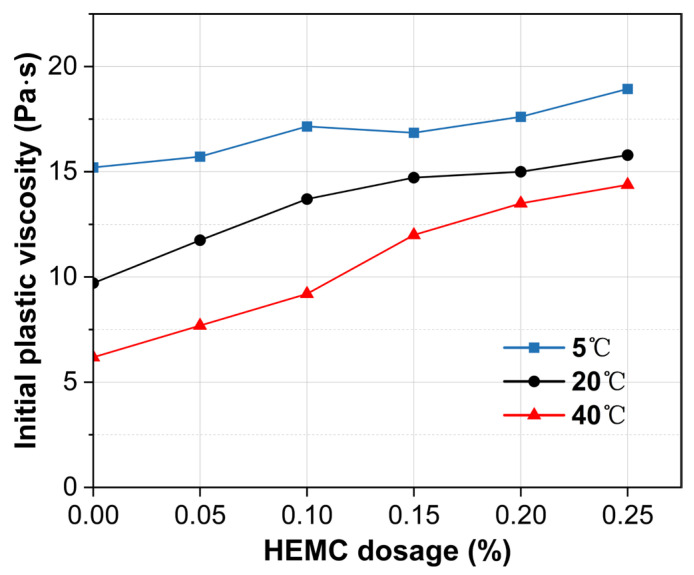
Initial plastic viscosity (at 10 min) of mortars with different HEMC dosages at 5 °C, 20 °C, and 40 °C.

Figure 8 presents the initial (10 min) plastic viscosity under the same conditions. Plastic viscosity increases monotonically with HEMC dosage, with the magnitude of increase rising with temperature. Specifically, raising HEMC from 0% to 0.25% augments viscosity by 24.5% at 5 °C, 62.7% at 20 °C, and 132.7% at 40 °C. Across all HEMC dosages, temperature exerts a pronounced influence on initial plastic viscosity. For the mortar without HEMC, the viscosity difference between 5 and 40 °C was 145%. Although this gap narrows as HEMC dosage increases, a disparity of approximately 30% still remains at the 0.25% dosage. This trend is primarily attributed to the temperature-sensitive viscosity of the liquid phase; both pure water and HEMC solutions become inherently more viscous at lower temperatures, which in turn elevates the plastic viscosity of mortar.

**Figure 9:**
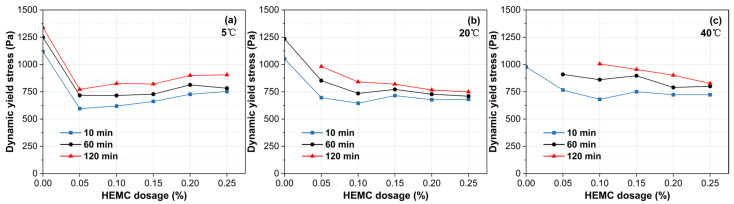
Evolution of dynamic yield stress over time for mortars with different HEMC dosages at: (**a**) 5 °C, (**b**) 20 °C, (**c**) 40 °C.

Figure 9 shows the development of dynamic yield stress over 120 min. Overall, the dynamic yield stress increases continuously over time, but the rate of this increase is dependent on both temperature and HEMC dosage. At 5 °C, the dynamic yield stress grows slowly across all HEMC levels, with the rate of increase consistently below 2.0 Pa/min. At 20 °C, this growth is moderated by HEMC addition. For mortars with less than 0.15% HEMC, the rate of increase ranged from 1.7 to 3.7 Pa/min, whereas at dosages of 0.15% and above, it stabilized at a low value of less than 1.0 Pa/min. As expected, the growth rates at 40 °C were higher than at lower temperatures. Nevertheless, a clear retarding effect was observed as HEMC content increased, with the rate progressively decreasing until it fell below 1.0 Pa/min at the 0.25% dosage.

**Figure 10:**
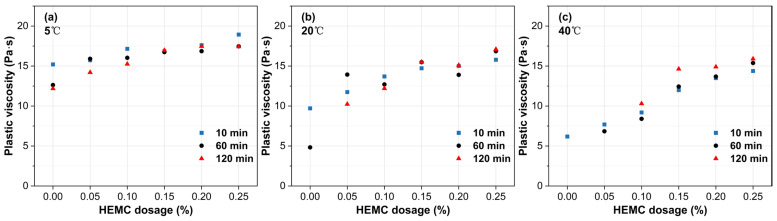
Evolution of plastic viscosity over time for mortars with different HEMC dosages at: (**a**) 5 °C, (**b**) 20 °C, (**c**) 40 °C.

The evolution of plastic viscosity over time reveals distinct trends at different temperatures (Figure 10). At 5 °C, a slight decrease in viscosity was observed. This decrease, likely attributable to the heat of hydration raising the mortar’s actual temperature, was 19.7% for the mortar without HEMC, while mortars containing HEMC declined by 5% to 15%. At 20 °C, the mortar without HEMC exhibited a sharp viscosity drop of 50.5% at 60 min, primarily due to bleeding and the resulting reduction in effective solid concentration under shear. In contrast, mortars containing 0.15% HEMC or more showed excellent stability, with viscosity fluctuations contained within ±15% of their initial values, maintaining a steady range between 14 and 17 Pa·s. At 40 °C, while low-dosage mortars set prematurely, those with sufficient HEMC showed a slight increase in plastic viscosity over time, with the total increase remaining below 22% over 120 min.

### 3.3. Microstructural Evolution by UPV

**Figure 11:**
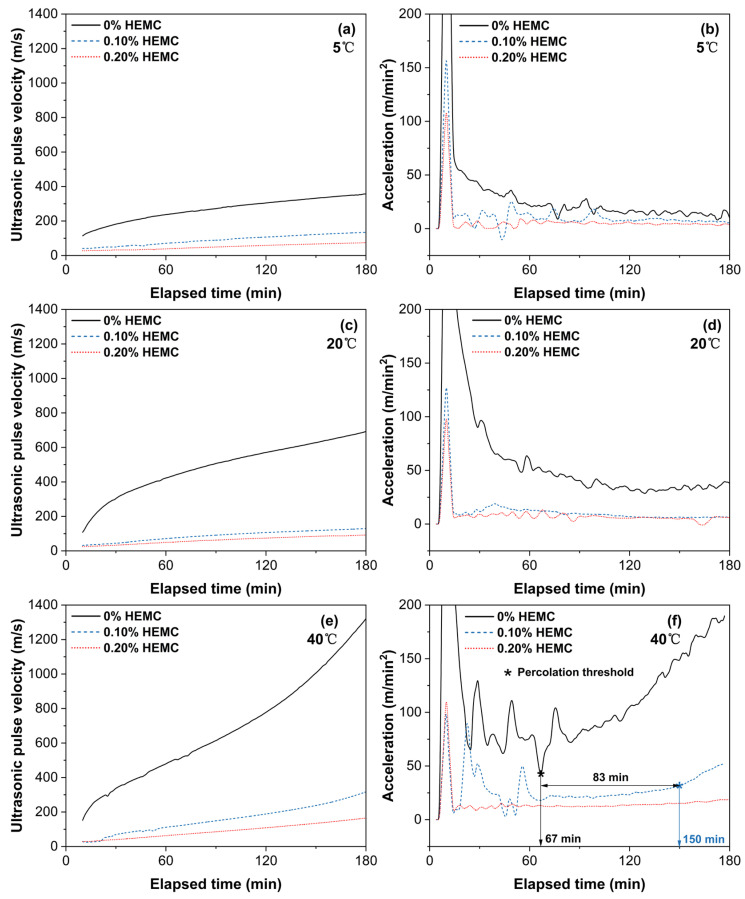
Ultrasonic pulse velocity (UPV) and acceleration evolution over 180 min for mortars with 0%, 0.10%, and 0.20% HEMC at different temperatures: (**a**) 5 °C, UPV; (**b**) 5 °C, acceleration; (**c**) 20 °C, UPV; (**d**) 20 °C, acceleration; (**e**) 40 °C, UPV; (**f**) 40 °C, acceleration.

To investigate how HEMC affects early microstructural development, UPV was continuously monitored for 180 min (Figure 11). The initial UPV of HEMC-modified mortars was significantly lower than that of the mortar without HEMC, likely due to air entrainment by HEMC that impedes ultrasonic transmission. Over time, UPV increased in all samples, indicating progressive microstructural development. At each temperature, the mortar without HEMC showed the fastest UPV growth, while that of the 0.20% HEMC mortar was the slowest. Increasing temperature accelerated UPV development across all mixtures. At 5 °C and 20 °C, all samples exhibited a steady, nearly linear increase in UPV over the 180 min period. By contrast, at 40 °C the mortar without HEMC reached its solid-phase percolation threshold at 67 min—evidenced by a sharp acceleration spike and sudden UPV surge—as hydration exited its dormant period and a continuous solid network formed [36]. The characteristic percolation transition in the 0.10% HEMC mortar was delayed to 150 min—an 83 min lag—while no such transition occurred within 180 min for the 0.20% HEMC mortar. This confirms that HEMC prolongs the dormant period of cement hydration and retards solid-network formation in a dosage-dependent manner.

**Figure 12:**
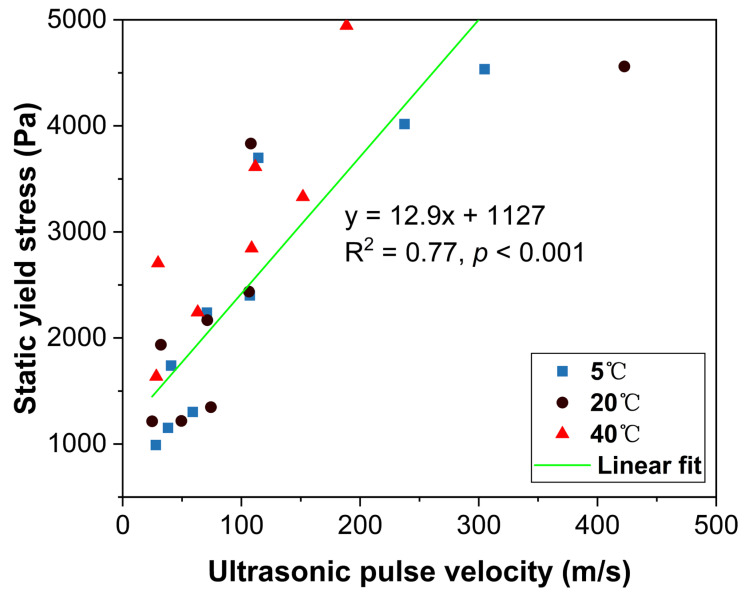
Correlation between static yield stress and ultrasonic pulse velocity measured at different elapsed times and temperatures.

To quantitatively link the macroscopic rheological properties with the underlying microstructural state, the static yield stress is plotted against the corresponding UPV value in Figure 12. A statistically significant positive correlation was observed between static yield stress and UPV, as confirmed by linear regression analysis. The relationship is described by the equation y = 12.9x + 1127, where y represents the static yield stress (Pa) and x is the UPV value (m/s). The Pearson correlation coefficient (r = 0.88) and coefficient of determination (R^2^ = 0.77) indicate a moderate-to-strong correlation with high statistical significance (*p* < 0.001). Although some scatter is observed across the data points, particularly when comparing different temperatures, the overall trend consistently supports the logical link between rheological resistance and ultrasonic propagation. This finding suggests that UPV can serve as a reliable, though not exclusive, indicator of early structural evolution in mortar.

### 3.4. XRD Analysis

**Figure 13:**
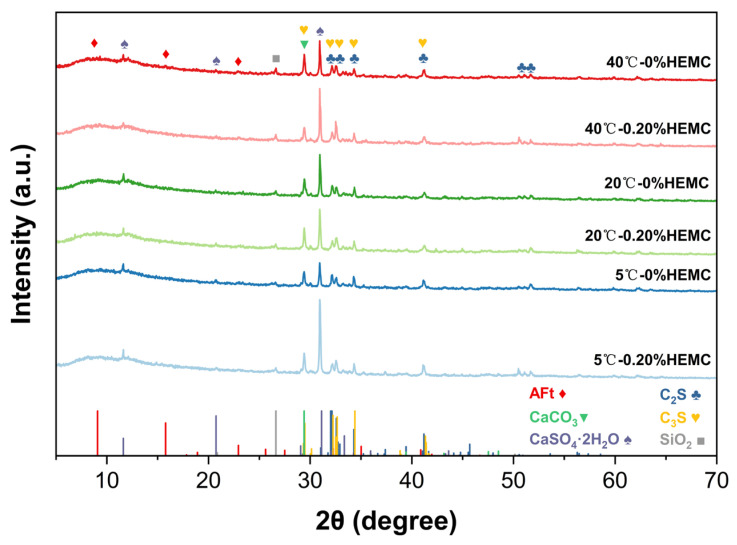
XRD patterns of cement pastes after 120 min of hydration at 5, 20, and 40 °C with 0% and 0.20% HEMC.

Figure 13 presents the XRD patterns of cement pastes after 120 min of hydration at 5, 20, and 40 °C with HEMC dosages of 0% and 0.20%. XRD analysis identified the main crystalline phases as tricalcium silicate (C_3_S), dicalcium silicate (C_2_S), calcite (CaCO_3_), gypsum (CaSO_4_·2H_2_O), quartz (SiO_2_), and ettringite (AFt). These phases are consistent with the raw materials: C_3_S, C_2_S, and gypsum originate from unhydrated clinker; calcite derives from limestone filler; quartz originates from unhydrated cement and fly ash; and AFt corresponds to early hydration products. The AFt peaks are broad and weak, indicating low crystallinity and content. The overall similarity in peak positions and intensities across all samples suggests that neither temperature nor HEMC addition significantly altered the crystalline phase within 120 min, reflecting the relatively low hydration degree at this stage.

No distinct reflections of portlandite (CH) or calcium silicate hydrate (C–S–H) were detected. This is reasonable, since CH may be present only in small amounts at 120 min, while C–S–H is largely amorphous and thus difficult to identify by conventional XRD. The absence of clear peaks therefore reflects the method’s limitation rather than the absence of these phases. To further verify the presence of amorphous hydration products and to characterize the microstructural features, complementary FTIR and SEM–EDS analyses were conducted.

### 3.5. FTIR Analysis

**Figure 14:**
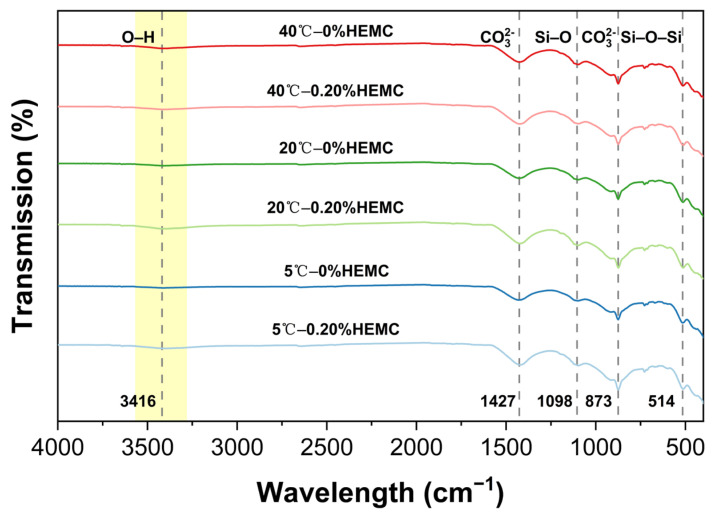
FTIR spectra of cement pastes after 120 min of hydration at 5, 20, and 40 °C with 0% and 0.20% HEMC.

FTIR spectra of cement pastes with 0% and 0.20% HEMC after 120 min of hydration at 5, 20, and 40 °C are shown in Figure 14. All samples exhibited characteristic absorption bands at 514, 873, 1098, 1427, and 3416 cm^−1^. The bands at 514 and 1098 cm^−1^ correspond to Si–O–Si bending and Si–O asymmetric stretching, confirming the presence of early hydration products, mainly C–S–H gels. The peaks at 873 and 1427 cm^−1^ are attributed to CO_3_^2−^ vibrations, primarily originating from the limestone filler. The nearly identical band positions across all samples indicate that neither temperature nor HEMC addition altered the main types of hydration products at this age.

The main difference between the spectra was observed at ~3416 cm^−1^, where a broad and weak O–H stretching band appeared slightly stronger in HEMC-modified pastes. This enhancement indicates additional hydrogen bonding from hydroxyl groups in HEMC and retained water molecules, suggesting that HEMC mainly influences the water state during early hydration rather than altering the composition of hydration products.

### 3.6. SEM-EDS Analysis

**Figure 15:**
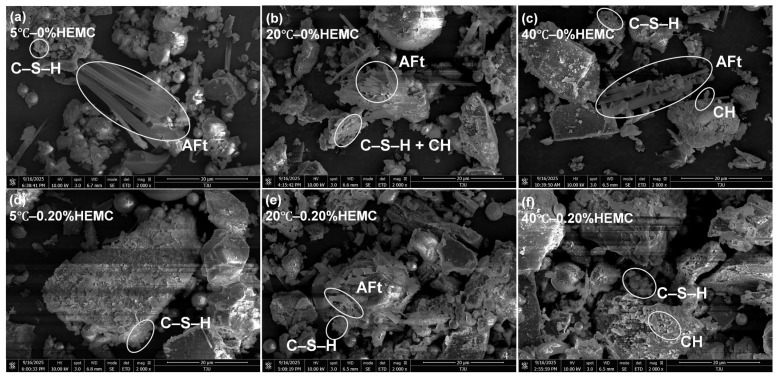
SEM micrographs of mortars after 120 min of hydration: (**a**) 5 °C—0% HEMC; (**b**) 20 °C—0% HEMC; (**c**) 40 °C—0% HEMC; (**d**) 5 °C—0.20% HEMC; (**e**) 20 °C—0.20% HEMC; (**f**) 40 °C—0.20% HEMC. AFt, C–S–H, and CH were identified based on morphological features and EDS analyses.

Figure 15 shows the SEM images of mortars with 0% and 0.20% HEMC after 120 min of hydration at 5 °C, 20 °C, and 40 °C. Representative elemental compositions of AFt, C–S–H, and CH, identified from selected EDS point analyses, are summarized in Table 6. In general, all samples exhibited a relatively low degree of hydration, and the fly ash particles remained largely unreacted at this stage. AFt and C–S–H were identified as the predominant hydration products across all conditions, whereas CH was detected only at 40 °C. At 5 °C, hydration was relatively limited, resulting in loose microstructures mainly composed of amorphous C–S–H with small amounts of AFt. At 20 °C, more AFt needles coexisted with dense C–S–H networks and minor CH plates, indicating more advanced hydration compared to 5 °C. At 40 °C, the microstructure was more compact, with abundant AFt and clustered C–S–H, accompanied by a small amount of hexagonal plate-like CH, consistent with the accelerated hydration revealed by UPV.

In HEMC-modified mortars, the overall morphology was less compact, with smaller and less crystalline hydrates. AFt was still present at all temperatures, but its abundance appeared lower than in mortars without HEMC, while C–S–H was finer and more uniformly distributed. CH formation was notably suppressed by HEMC, with only trace amounts detected at 40 °C. These observations confirm that HEMC retards hydration and alters the development of crystalline phases, favoring the formation of a gel-rich microstructure. Such microstructural modifications are in line with the rheological and UPV results, which demonstrated delayed structural buildup and stabilized performance under different temperature conditions.

## 4. Conclusions

This study systematically investigated the temperature-dependent effects of a high-viscosity (75,000 mPa·s) HEMC on the rheological properties and early-stage microstructural evolution of robotic plastering mortars at 5 °C, 20 °C, and 40 °C. The key findings are as follows:

1. The incorporation of HEMC consistently reduced the initial static yield stress while monotonically increasing the plastic viscosity, with the viscosity-enhancing effect being more pronounced at higher temperatures. The impact on initial dynamic yield stress was less significant, with the effect stabilizing around 625–750 Pa at a dosage of 0.05% for 5 °C and 0.10% for 20 °C and 40 °C.

2. Temperature strongly modulated rheological evolution: at 40 °C, the buildup of static and dynamic yield stresses was markedly accelerated, whereas at 5 °C, structural development was considerably delayed. HEMC addition effectively counteracted these temperature effects by retarding hydration-induced stiffening and stabilizing plastic viscosity.

3. UPV measurements demonstrated that HEMC prolonged the dormant period and delayed solid-phase percolation in a dosage-dependent manner. Notably, at 40 °C, a 0.10% HEMC dosage delayed the solid-phase percolation threshold to 150 min (an 83 min lag compared to the control group), and a 0.20% dosage prevented this transition from occurring within the 180 min test period. A statistically significant moderate-to-strong correlation (r = 0.88, R^2^ = 0.77, *p* < 0.001) between static yield stress and UPV confirmed the link between rheological resistance and microstructural densification.

4. Complementary XRD, FTIR, and SEM–EDS analyses revealed that within the first 120 min of hydration, HEMC suppressed the formation of crystalline portlandite, reduced AFt abundance, and promoted a gel-rich microstructure with finer C–S–H. These findings corroborate the rheological and ultrasonic observations, highlighting HEMC’s role in moderating temperature-driven hydration kinetics.

5. Based on the combined results, the recommended HEMC dosage ranges for ensuring stable rheological properties in robotic plastering mortars are 0.05–0.10% at 5 °C, 0.10–0.15% at 20 °C, and 0.15–0.20% at 40 °C.

Future studies will extend this work to sub-zero environments to evaluate mortar performance in cold-region construction.

## Figures and Tables

**Table 1:** Chemical composition of cement and fly ash (wt, %).

Binder	SiO_2_	Al_2_O_3_	Fe_2_O_3_	CaO	MgO	Na_2_O	K_2_O	SO_3_	CO_3_^2−^	Cl^−^
Cement	22.33	6.94	3.52	57.64	1.97	0.25	0.78	2.43	3.6	0.024
Fly ash	44.84	40.94	3.38	0.34	2.93	0.57	0.66	0.65	—	—

**Table 2:** Gradation of manufactured sand.

Sieve Size (mm)	4.75	2.36	1.18	0.6	0.3	0.15	0.075	0
Individual sieve residue (%)	0	10.48	23.75	26.20	25.15	12.07	1.87	0.28
Cumulative sieve residue (%)	0	10.48	34.23	60.43	85.59	97.66	99.53	99.81

**Table 3:** Physical properties of manufactured sand.

Maximum Size (mm)	Apparent Density (kg/m^3^)	Loose Bulk Density (kg/m^3^)	Loose Bulk Porosity (%)	Fineness Modulus
2.36	2830	1570	45	2.88

**Table 4:** Mix proportions and temperature conditions.

Specimens	Cement (g)	Fly Ash (g)	Sand (g)	Limestone Powder (g)	HEMC (g)	Temperature (°C)
0% HEMC	200	50	675	75	0	5/20/40
0.05% HEMC	200	50	675	75	0.125	
0.10% HEMC	200	50	675	75	0.250	
0.15% HEMC	200	50	675	75	0.375	
0.20% HEMC	200	50	675	75	0.500	
0.25% HEMC	200	50	675	75	0.625	

**Table 5:** Repeatability test results for 20 °C, 0.05% HEMC, 10 min mortar sample.

Parameter	Replicate 1	Replicate 2	Replicate 3	Mean	Standard Deviation	Coefficient of Variation (CV)
Static yield stress (Pa)	2897	2857	2826	2860	36.1	1.30%
Dynamic yield stress (Pa)	696	738	776	737	40	5.40%
Plastic viscosity (Pa·s)	11.7	11.6	11.3	11.5	0.2	1.80%

**Table 6:** Representative EDS elemental compositions (atomic %) of typical hydration products identified in SEM micrographs after 120 min.

Phase	O (at%)	Ca (at%)	Si (at%)	Al (at%)	S (at%)	Other (at%)
AFt	31.8	8.38	3.12	1.5	3.76	C 49.46; Mg 1.26; K 0.72
C–S–H	47.01	20.02	21.51	6.59	—	Fe 2.44; K 1.50; Mg 0.93
CH	—	88.07	7.32	0.71	—	Fe 3.50; K 0.40

## Data Availability

The original contributions presented in this study are included in the article/Supplementary Material. Further inquiries can be directed to the corresponding author.

## Supplementary Materials

The following supporting information can be downloaded at: https://www.mdpi.com/article/10.3390/ma18204664/s1, Table S1: Rheological model fitting results for dynamic flow curves at different temperatures and HEMC dosages.
