# Supplementary material for: Temperature-Dependent Effects of Hydroxyethyl Methyl Cellulose on Rheological Properties and Microstructural Evolution of Robotic Plastering Mortars
Source: Materials (Basel). 2025 Oct 10;18(20):4664. doi: 10.3390/ma18204664 (PMC12565420; doi:10.3390/ma18204664)
Supplement: Supplementary file 1 [file materials-18-04664-s001.zip › materials-3840842-supplementary.pdf]

Table S1. Rheological model fitting results for dynamic flow curves at different temperatures and HEMC dosages.

Specimens	Elapsed time (min)	Bingham model			Modified Bingham model			Herschel-Bulkley model		
		Equation	R^2	AIC	Equation	R^2	AIC	Equation	R^2	AIC
	10	$\tau = 1119.00 + 15.20 * \dot{\gamma}$	0.97	1099	$\tau = 1123.50 + 14.86 * \dot{\gamma} + 0.0046 * \dot{\gamma}^2$	0.97	1101	$\tau = 1123.51 + 14.46 * \dot{\gamma}^{1.01}$	0.97	1101
5-0% HEMC	60	$\tau = 1249.05 + 12.62 * \dot{\gamma}$	0.96	1104	$\tau = 1289.61 + 9.54 * \dot{\gamma} + 0.0415 * \dot{\gamma}^2$	0.96	1094	$\tau = 1303.94 + 5.35 * \dot{\gamma}^{1.19}$	0.96	1097
	120	$\tau = 1339.60 + 12.18 * \dot{\gamma}$	0.93	1174	$\tau = 1392.38 + 8.17 * \dot{\gamma} + 0.0540 * \dot{\gamma}^2$	0.94	1163	$\tau = 1414.40 + 3.26 * \dot{\gamma}^{1.29}$	0.94	1163
	10	$\tau = 595.92 + 15.72 * \dot{\gamma}$	0.99	956	$\tau = 642.34 + 12.16 * \dot{\gamma} + 0.0481 * \dot{\gamma}^2$	0.99	909	$\tau = 665.09 + 6.53 * \dot{\gamma}^{1.19}$	0.99	909
5-0.05% HEMC	60	$\tau = 717.51 + 15.90 * \dot{\gamma}$	0.99	968	$\tau = 734.12 + 14.62 * \dot{\gamma} + 0.0172 * \dot{\gamma}^2$	0.99	965	$\tau = 743.62 + 11.86 * \dot{\gamma}^{1.06}$	0.99	966
	120	$\tau = 772.09 + 14.20 * \dot{\gamma}$	0.98	1009	$\tau = 766.92 + 14.60 * \dot{\gamma} - 0.0053 * \dot{\gamma}^2$	0.98	1011	$\tau = 765.55 + 15.32 * \dot{\gamma}^{0.98}$	0.98	1011
	10	$\tau = 618.91 + 17.15 * \dot{\gamma}$	0.99	969	$\tau = 581.24 + 20.03 * \dot{\gamma} - 0.0390 * \dot{\gamma}^2$	0.99	943	$\tau = 1253.06 + 23.49 * \dot{\gamma}^{-18.05}$	0.00	1630
5-0.10% HEMC	60	$\tau = 716.03 + 16.02 * \dot{\gamma}$	0.99	1024	$\tau = 782.85 + 10.90 * \dot{\gamma} + 0.0693 * \dot{\gamma}^2$	0.99	960	$\tau = 807.98 + 4.75 * \dot{\gamma}^{1.2752}$	0.99	968
	120	$\tau = 825.63 + 15.25 * \dot{\gamma}$	0.99	995	$\tau = 812.62 + 16.25 * \dot{\gamma} - 0.0135 * \dot{\gamma}^2$	0.99	994	$\tau = 805.37 + 18.87 * \dot{\gamma}^{0.95}$	0.99	995
	10	$\tau = 662.15 + 16.85 * \dot{\gamma}$	0.98	1106	$\tau = 546.21 + 25.73 * \dot{\gamma} - 0.1202 * \dot{\gamma}^2$	0.99	971	$\tau = 1285.18 + 46.60 * \dot{\gamma}^{-36.89}$	0.00	1627
5-0.15% HEMC	60	$\tau = 728.29 + 16.74 * \dot{\gamma}$	0.99	1034	$\tau = 653.22 + 22.49 * \dot{\gamma} - 0.0778 * \dot{\gamma}^2$	0.99	955	$\tau = 1347.43 + 34.22 * \dot{\gamma}^{-26.67}$	0.00	1624
	120	$\tau = 821.46 + 16.96 * \dot{\gamma}$	0.98	1058	$\tau = 758.35 + 21.79 * \dot{\gamma} - 0.0654 * \dot{\gamma}^2$	0.99	1018	$\tau = 1448.55 + 32.12 * \dot{\gamma}^{-25.33}$	0.00	1628
	10	$\tau = 727.16 + 17.60 * \dot{\gamma}$	0.98	1115	$\tau = 614.79 + 26.21 * \dot{\gamma} - 0.1165 * \dot{\gamma}^2$	0.99	1004	$\tau = 1378.14 + 47.61 * \dot{\gamma}^{-37.74}$	0.00	1639
5-0.20% HEMC	60	$\tau = 813.58 + 16.85 * \dot{\gamma}$	0.97	1117	$\tau = 693.66 + 26.04 * \dot{\gamma} - 0.1244 * \dot{\gamma}^2$	0.99	982	$\tau = 1436.86 + 47.21 * \dot{\gamma}^{-38.04}$	0.00	1628
	120	$\tau = 899.81 + 17.45 * \dot{\gamma}$	0.98	1100	$\tau = 801.15 + 25.01 * \dot{\gamma} - 0.1023 * \dot{\gamma}^2$	0.99	1013	$\tau = 1545.23 + 43.66 * \dot{\gamma}^{-34.95}$	0.00	1637
	10	$\tau = 752.37 + 18.93 * \dot{\gamma}$	0.98	1091	$\tau = 541.66 + 27.41 * \dot{\gamma} - 0.1148 * \dot{\gamma}^2$	0.99	950	$\tau = 1352.40 + 47.20 * \dot{\gamma}^{-36.49}$	0.00	1659
5-0.25% HEMC	60	$\tau = 782.66 + 17.46 * \dot{\gamma}$	0.98	1089	$\tau = 564.85 + 26.48 * \dot{\gamma} - 0.1222 * \dot{\gamma}^2$	0.99	906	$\tau = 1328.28 + 47.41 * \dot{\gamma}^{-37.41}$	0.00	1637
	120	$\tau = 905.83 + 17.42 * \dot{\gamma}$	0.96	1188	$\tau = 629.83 + 30.90 * \dot{\gamma} - 0.1826 * \dot{\gamma}^2$	0.99	961	$\tau = 1449.96 + 68.08 * \dot{\gamma}^{-51.43}$	0.00	1639
20-0% HEMC	10	$\tau = 1051.04 + 9.70 * \dot{\gamma}$	0.85	1238	$\tau = 1214.34 - 2.70 * \dot{\gamma} + 0.1672 * \dot{\gamma}^2$	0.92	1143	$\tau = 1201.32 + 0.02 * \dot{\gamma}^{2.43}$	0.93	1140
	60	$\tau = 1235.51 + 4.82 * \dot{\gamma}$	0.52	1272	$\tau = 1298.20 + 0.06 * \dot{\gamma} + 0.0641 * \dot{\gamma}^2$	0.55	1266	$\tau = 1300.53 + 0.05 * \dot{\gamma}^{2.04}$	0.55	1266
	10	$\tau = 696.40 + 11.74 * \dot{\gamma}$	0.99	949	$\tau = 684.82 + 14.63 * \dot{\gamma} - 0.0120 * \dot{\gamma}^2$	0.99	948	$\tau = 680.71 + 16.52 * \dot{\gamma}^{0.95}$	0.99	949
20-0.05% HEMC	60	$\tau = 852.13 + 13.93 * \dot{\gamma}$	0.98	1059	$\tau = 889.72 + 11.05 * \dot{\gamma} + 0.0390 * \dot{\gamma}^2$	0.98	1048	$\tau = 907.32 + 6.41 * \dot{\gamma}^{1.17}$	0.98	1049
	120	$\tau = 982.18 + 10.23 * \dot{\gamma}$	0.98	962	$\tau = 996.99 + 9.10 * \dot{\gamma} + 0.0153 * \dot{\gamma}^2$	0.98	960	$\tau = 1009.16 + 6.27 * \dot{\gamma}^{1.10}$	0.98	958

20-0.10% HEMC	10	$\tau = 645.31 + 11.74 * \dot{\gamma}$	0.98	954	$\tau = 622.47 + 13.48 * \dot{\gamma} - 0.0233 * \gamma^2$	0.99	945	$\tau = 1083.14 + 13.99 * \gamma^{-12.70}$	0.00	1537
	60	$\tau = 735.37 + 12.65 * \dot{\gamma}$	0.98	993	$\tau = 773.23 + 9.77 * \dot{\gamma} + 0.0387 * \gamma^2$	0.98	972	$\tau = 789.65 + 5.43 * \gamma^{1.19}$	0.98	974
	120	$\tau = 840.84 + 12.17 * \dot{\gamma}$	0.98	977	$\tau = 858.32 + 10.84 * \dot{\gamma} + 0.0179 * \gamma^2$	0.98	974	$\tau = 867.23 + 8.23 * \gamma^{1.08}$	0.98	974
20-0.15% HEMC	10	$\tau = 715.30 + 13.72 * \dot{\gamma}$	0.98	1004	$\tau = 649.28 + 19.78 * \dot{\gamma} - 0.0684 * \gamma^2$	0.99	929	$\tau = 1259.56 + 30.08 * \gamma^{-24.06}$	0.00	1588
	60	$\tau = 772.03 + 15.48 * \dot{\gamma}$	0.98	1020	$\tau = 693.39 + 21.51 * \dot{\gamma} - 0.0815 * \gamma^2$	0.99	915	$\tau = 1344.59 + 32.46 * \gamma^{-25.96}$	0.00	1603
	120	$\tau = 819.96 + 15.48 * \dot{\gamma}$	0.99	959	$\tau = 823.19 + 15.24 * \dot{\gamma} + 0.0033 * \gamma^2$	0.99	961	$\tau = 816.31 + 16.10 * \gamma^{0.99}$	0.99	961
20-0.20% HEMC	10	$\tau = 676.95 + 15.00 * \dot{\gamma}$	0.96	1130	$\tau = 538.83 + 23.50 * \dot{\gamma} - 0.1414 * \gamma^2$	0.99	922	$\tau = 1161.43 + 47.95 * \gamma^{-40.43}$	0.00	1570
	60	$\tau = 727.95 + 13.86 * \dot{\gamma}$	0.96	1136	$\tau = 588.12 + 24.49 * \dot{\gamma} - 0.1431 * \gamma^2$	0.99	939	$\tau = 1244.74 + 48.27 * \gamma^{-41.23}$	0.00	1587
	120	$\tau = 766.86 + 15.07 * \dot{\gamma}$	0.98	1082	$\tau = 660.87 + 23.13 * \dot{\gamma} - 0.1085 * \gamma^2$	0.99	940	$\tau = 1328.50 + 41.51 * \gamma^{-33.61}$	0.00	1608
20-0.25% HEMC	10	$\tau = 680.80 + 15.79 * \dot{\gamma}$	0.97	1073	$\tau = 586.09 + 21.04 * \dot{\gamma} - 0.0982 * \gamma^2$	0.99	970	$\tau = 1190.69 + 38.52 * \gamma^{-31.45}$	0.00	1572
	60	$\tau = 709.68 + 16.85 * \dot{\gamma}$	0.98	1090	$\tau = 592.04 + 25.86 * \dot{\gamma} - 0.1220 * \gamma^2$	0.99	910	$\tau = 1332.72 + 46.08 * \gamma^{-36.60}$	0.00	1627
	120	$\tau = 849.14 + 15.09 * \dot{\gamma}$	0.95	1187	$\tau = 672.95 + 28.59 * \dot{\gamma} - 0.1828 * \gamma^2$	0.99	957	$\tau = 1407.30 + 66.16 * \gamma^{-20.73}$	0.00	1601
40-0% HEMC	10	$\tau = 978.28 + 6.18 * \dot{\gamma}$	0.74	1204	$\tau = 1067.08 - 0.56 * \dot{\gamma} + 0.0909 * \gamma^2$	0.79	1177	$\tau = 1065.92 + 0.04 * \gamma^{2.16}$	0.79	1177
40-0.05% HEMC	10	$\tau = 766.66 + 7.69 * \dot{\gamma}$	0.93	1035	$\tau = 765.96 + 7.74 * \dot{\gamma} - 0.0007 * \gamma^2$	0.93	1037	$\tau = 770.82 + 7.01 * \gamma^{1.02}$	0.93	1037
	60	$\tau = 909.24 + 6.84 * \dot{\gamma}$	0.87	1102	$\tau = 997.11 + 0.10 * \dot{\gamma} + 0.0911 * \gamma^2$	0.92	1038	$\tau = 998.79 + 0.09 * \gamma^{2.00}$	0.92	1039
40-0.10% HEMC	10	$\tau = 681.41 + 9.15 * \dot{\gamma}$	0.95	948	$\tau = 724.20 + 2.90 * \dot{\gamma} + 0.0438 * \gamma^2$	0.96	905	$\tau = 734.95 + 0.73 * \gamma^{1.48}$	0.96	906
	60	$\tau = 861.10 + 8.35 * \dot{\gamma}$	0.89	1067	$\tau = 940.61 + 0.31 * \dot{\gamma} + 0.0814 * \gamma^2$	0.93	997	$\tau = 943.26 + 0.10 * \gamma^{1.96}$	0.93	997
	120	$\tau = 1006.27 + 10.28 * \dot{\gamma}$	0.76	1146	$\tau = 1393.09 + 2.48 * \dot{\gamma} + 0.0377 * \gamma^2$	0.77	1141	$\tau = 1401.42 + 0.64 * \gamma^{1.4}$	0.78	1138
40-15% HEMC	10	$\tau = 750.74 + 11.99 * \dot{\gamma}$	0.96	1114	$\tau = 629.65 + 22.27 * \dot{\gamma} - 0.1256 * \gamma^2$	0.99	972	$\tau = 1231.06 + 42.85 * \gamma^{-35.73}$	0.00	1558
	60	$\tau = 897.22 + 12.43 * \dot{\gamma}$	0.98	1073	$\tau = 856.90 + 18.52 * \dot{\gamma} - 0.0418 * \gamma^2$	0.98	1061	$\tau = 1467.75 + 23.174 * \gamma^{-19.93}$	0.00	1603
	120	$\tau = 955.15 + 14.63 * \dot{\gamma}$	0.98	1052	$\tau = 954.94 + 15.65 * \dot{\gamma} - 0.0002 * \gamma^2$	0.98	1054	$\tau = 965.80 + 13.90 * \gamma^{1.02}$	0.98	1053
40-0.20% HEMC	10	$\tau = 723.36 + 13.47 * \dot{\gamma}$	0.97	967	$\tau = 559.34 + 14.34 * \dot{\gamma} - 0.0655 * \gamma^2$	0.99	861	$\tau = 976.60 + 23.32 * \gamma^{-20.32}$	0.00	1479
	60	$\tau = 789.80 + 13.68 * \dot{\gamma}$	0.97	1065	$\tau = 747.08 + 7.04 * \dot{\gamma} + 0.0893 * \gamma^2$	0.99	972	$\tau = 771.66 + 1.96 * \gamma^{1.44}$	0.99	976
	120	$\tau = 901.73 + 14.88 * \dot{\gamma}$	0.97	1092	$\tau = 999.20 + 6.99 * \dot{\gamma} + 0.0793 * \gamma^2$	0.98	1040	$\tau = 1021.86 + 2.12 * \gamma^{1.41}$	0.98	1046
40-0.25% HEMC	10	$\tau = 722.63 + 14.38 * \dot{\gamma}$	0.95	1204	$\tau = 593.30 + 30.89 * \dot{\gamma} - 0.1964 * \gamma^2$	0.99	958	$\tau = 1388.35 + 70.49 * \gamma^{-11.02}$	0.00	1623
	60	$\tau = 800.56 + 15.38 * \dot{\gamma}$	0.96	1146	$\tau = 693.75 + 23.56 * \dot{\gamma} - 0.1108 * \gamma^2$	0.98	1076	$\tau = 1369.20 + 44.29 * \gamma^{-36.13}$	0.00	1604

120	$\tau = 826.85 + 15.89 * \dot{\gamma}$	0.98	1089	$\tau = 831.95 + 17.50 * \dot{\gamma} + 0.0052 * \dot{\gamma}^2$	0.98	1091	$\tau = 810.47 + 20.76 * \dot{\gamma}^{0.96}$	0.98	1090
